# Ferromagnetically filled carbon nano-onions: the key role of sulfur in dimensional, structural and electric control

**DOI:** 10.1098/rsos.170981

**Published:** 2018-01-17

**Authors:** D. Medranda, J. Borowiec, Xiao Zhang, S. Wang, K. Yan, J. Zhang, Y. He, S. Ivaturi, F. S. Boi

**Affiliations:** 1 College of Physical Science and Technology, Sichuan University, Chengdu, People's Republic of China; 2 Analytical and Testing Centre, Sichuan University, Chengdu, People's Republic of China; 3 School of Chemistry and Chemical Engineering, Huazhong University of Science and Technology, Wuhan, People's Republic of China

**Keywords:** ferromagnetic, sulfur, carbon onions

## Abstract

A key challenge in the fabrication of ferromagnetically filled carbon nano-onions (CNOs) is the control of their thickness, dimensions and electric properties. Up to now literature works have mainly focused on the encapsulation of different types of ferromagnetic materials including α-Fe, Fe_3_C, Co, FeCo, FePd_3_ and others within CNOs. However, no report has yet shown a suitable method for controlling both the number of shells, diameter and electric properties of the produced CNOs. Here, we demonstrate an advanced chemical vapour deposition approach in which the use of small quantities of sulfur during the pyrolysis of ferrocene allows for the control of (i) the diameter of the CNOs, (ii) the number of shells and (iii) the electric properties. We demonstrate the morphological, structural, electric and magnetic properties of these new types of CNOs by using SEM, XRD, TEM, HRTEM, EIS and VSM techniques.

## 1. Introduction

Carbon nano-onions (CNOs) are generally described as fullerene-like carbon structures consisting of concentrically arranged nested graphitic layers surrounding a C_60_ core. The number of CNO shells reported up to now is generally in the order of 10–50 and has been described more in general as consisting of 60N^2^ carbon atoms [1–10], with N being the number of carbon layers [1–9]. Thanks to the important chemical and physical properties, these structures have recently become an important focus of research which show promise for applications in aerospace as additive [9], energy storage as capacitors [10] and miniaturized fuel cells [8]. In particular, these structures have been reported to exhibit giant capacitance and high conductivity (10 S cm^−2^) [10]. In addition to these excellent properties, CNOs have been recently considered important candidates also for magnetic data storage applications as high-density recording media [11–16]. Indeed, due to the excellent chemical stability, these structures can be used as nano-sized containers for the encapsulation of specific materials of interest and their protection from oxidation. In the contest of high-density data storage devices, important attention has been posed on the encapsulation of ferromagnetic single crystals within CNOs [11–18]. Several types of ferromagnets have been encapsulated inside CNOs; these include Fe_3_C, Fe_7_C_3_, Fe_5_C_2_, Fe, Co, Ni, FeCo, CoFe_2_O_4_, FePd_3_ and others [11–18]. Thanks to the high magneto-crystalline anisotropy and/or the single magnetic domain critical size dimensionality these materials can indeed exhibit large coercivities and high magnetizations [13–16]. However, despite the important advancement, several critical challenges remain in order to translate filled CNOs into technological applications. One of these challenges is the control of CNO diameter and thickness (number of shells). The possibility of controlling these dimensional parameters is generally limited by the used metallocene-like synthesis precursors, which mainly contain C, H and metal elements. Interestingly, a recent report has shown that the use of Cl radicals can allow decreasing the thickness of CNOs produced in viscous boundary layer chemical vapour synthesis experiments involving the pyrolysis of ferrocene/dichlorobenzene mixtures [19]. However, due to the local etching dynamics of Cl radicals a dramatic decrease in the crystallinity and therefore an amorphization of the CNO layers was also reported. New synthesis approaches are therefore required in order to achieve a higher degree of synthesis control of these nanostructures and investigate their potential for future applications.

In this communication, we demonstrate an advanced synthesis approach which allows the achievement of such synthesis control. We find that the presence of small quantities of sulfur species in the pyrolysis of ferrocene can allow a high control of the diameter and thickness (number of shells) of CNOs. Surprisingly, the CNOs grown in the presence of such species are found to be encapsulated with unusual iron-carbide phases, namely Fe_7_C_3_ and Fe_5_C_2_, which have been previously reported in high-pressure studies [17,18]. Our results suggest that the presence of sulfur allows the trapping of these phases within CNOs and inhibits the formation of Fe_3_C crystals due to intrinsic local changes in the carbon diffusion/extrusion flux dynamics. The properties of these structures are characterized in detail by using numerous techniques: scanning electron microscopy (SEM), transmission electron microscopy (TEM), high resolution TEM (HRTEM), temperature-dependent X-ray diffraction (T-XRD), temperature-dependent vibrating sample magnetometry (T-VSM). In addition, the impedance properties of these structures have been characterized with electrochemical impedance spectroscopy (EIS) and compared with those of Fe_3_C-filled CNOs.

## 2. Experimental

Millimetre scale buckypapers of Fe_5_C_2_/Fe_7_C_3_-filled CNOs were obtained by sublimation and pyrolysis of 400 mg of ferrocene together with 2.5 mg of sulfur in an Ar flow of 11 ml min^−1^ on the top of Si/SiO_2_ substrates positioned inside a quartz tube reactor of length 1.5 m. A one-zone electric furnace was used for reaching the desired temperature of pyrolysis (temperature of 990°C). The used sublimation temperature was 700°C. The used reaction time was 10 min. A fast cooling method was used to bring the CNOs-buckypaper to room temperature. In this method, cooling times of approximately 20 min were achieved by removing the furnace along a rail system (quench). The Fe_5_C_2_/Fe_7_C_3_-filled CNOs-buckypaper was removed through the use of a permanent magnet from the upper-surface of aligned single-wall CNT films (see electronic supplementary material, figure S11 for SEM and TEM images of these films) grown on the Si/SiO_2_ substrates and from the first edge of the Si/SiO_2_ substrates (edge facing the Ar flow). See electronic supplementary material for additional information of the samples characterization. Additional experiments were also performed with higher quantities of ferrocene (see electronic supplementary material); however, the CNOs obtained in such conditions were found to be characterized by an hollow morphology and by an increased level of turbostratic arrangement, possibly due to the too high concentration of carbon present in the pyrolysis system which would then inhibit the homogeneous nucleation of CNOs within the reactor. Additionally, note that the use of higher quantities of sulfur led to a disappearance of the CNO morphology and the appearance of carbon fibre-like structures.

## 3. Results and discussion

**Figure 1.**
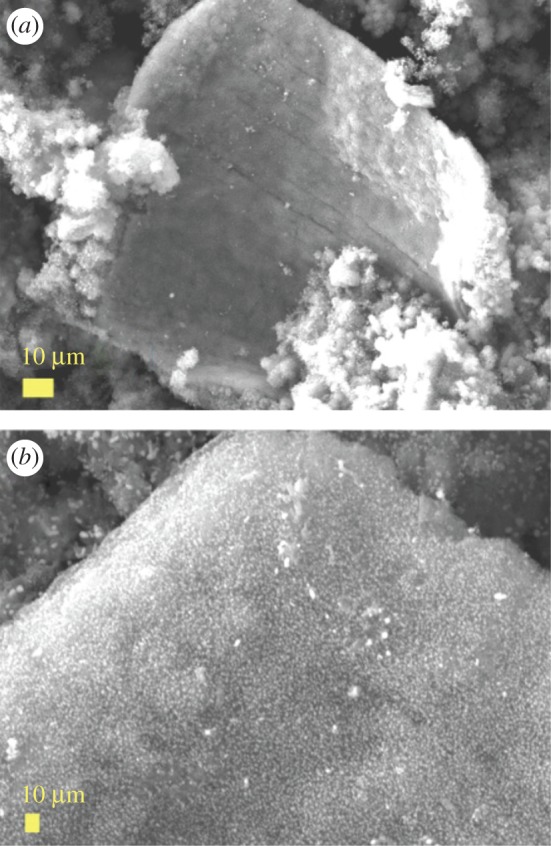
SEM micrographs (*a*,*b*) showing the surface morphology of a micrometre scale flake buckypaper of CNOs filled with Fe_5_C_2_/Fe_7_C_3_ crystals.

**Figure 2.**
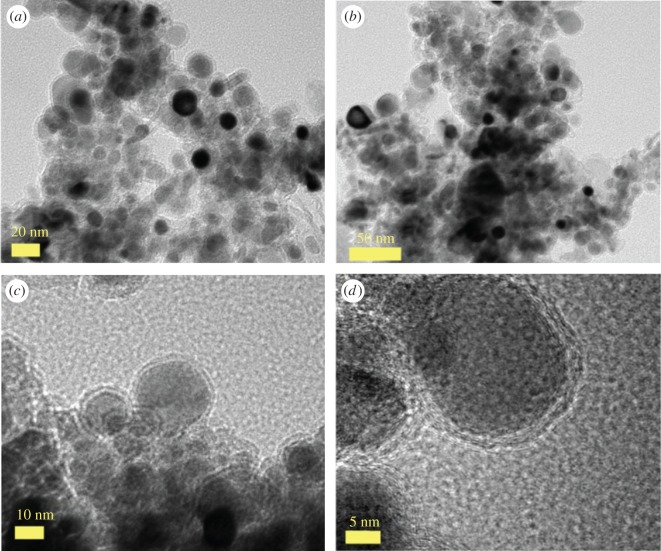
TEM micrographs (*a*-*d*) showing the cross-sectional morphology of the as grown Fe_5_C_2_/Fe_7_C_3_-filled CNOs.

The morphology of the as grown CNOs was firstly revealed by SEM measurements. A typical micrograph showing the surface morphology of a flake of the as grown CNOs-buckypaper is shown in figure 1*a*. A higher detail of such buckypaper is shown in figure 1*b*. In order to better visualize the cross-sectional morphology of individual CNOs comprised in the buckypaper, the use of TEM and HRTEM was considered. As shown in figure 2, these measurements revealed the presence of CNOs with a diameter in the order of 10–30 nm, much smaller than that reported in previous works based on the pyrolysis of only ferrocene (figure 2*a–d* and electronic supplementary material, figure S1 for statistical analyses). Additionally, we observe that the CNOs obtained in these conditions are characterized by a number of layers ranging from a minimum of 4 to a maximum of 10 (see electronic supplementary material, figures S1 and S2).

Such number of shells is much smaller than that reported in literature for CNOs produced with only ferrocene (10–50 layers) [13].

The attention was then turned on the structural properties of the produced CNOs. As shown in electronic supplementary material, figure S2 the presence of graphitic layers in the CNOs was confirmed by the observation of multiple graphitic peaks in the range between 16° and 27° 2*θ*. Furthermore, measurements performed in the angular range between 39° and 51° 2*θ* allowed for the identification of the magnetic phases encapsulated within the CNO core.

**Figure 3.**
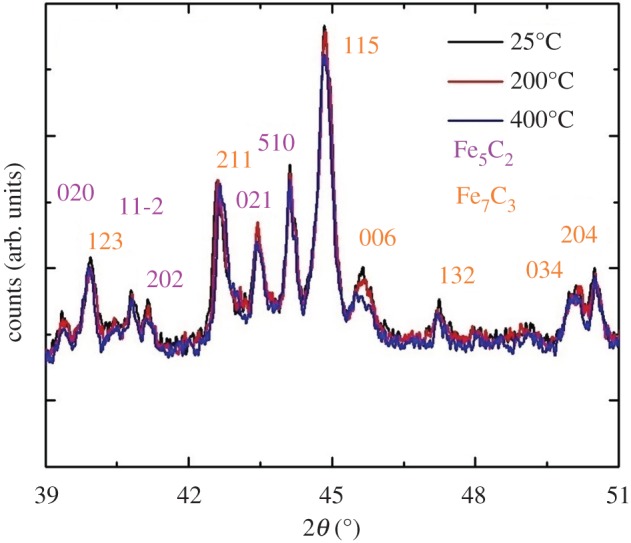
XRD diffractogram of the as grown CNOs-buckypaper filled with Fe_5_C_2_/Fe_7_C_3_ crystals.

**Figure 4.**
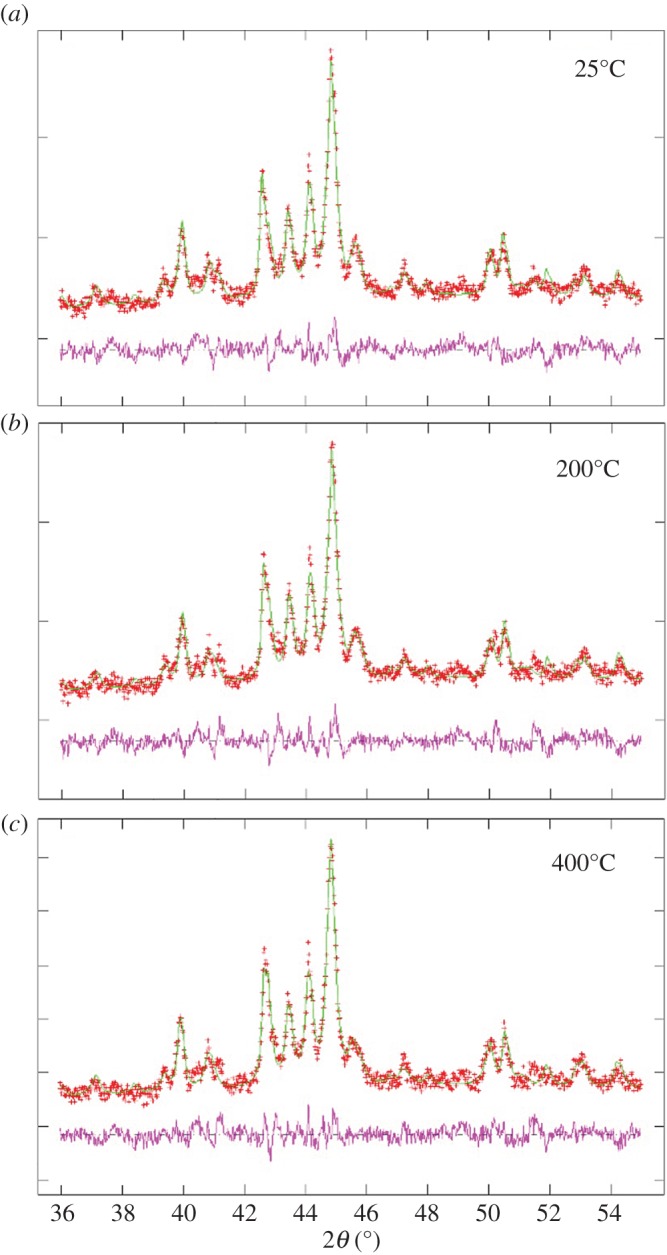
In (*a*–*c*), temperature-dependent XRD diffractograms (red crosses) and Rietveld refinements (green lines) of the as grown CNOs-buckypaper filled with Fe_5_C_2_/Fe_7_C_3_ crystals. The difference between the experimental data and the Rietveld refinement fittings is represented by the magenta line.

As shown in figure 3, surprisingly these measurements revealed the presence of two main phases which are generally observed only in high-pressure experiments, namely Fe_7_C_3_ and Fe_5_C_2_. This interpretation was confirmed by additional Rietveld refinement analyses shown in figure 4, which allowed the extraction of the following lattice parameters *a*: 0.1158 nm *b*: 0.458 nm and *c*: 0.506 nm for the Fe_5_C_2_ case and *a*: 0.454 nm, *b*: 0.690 nm and *c*: 0.119 nm for the Fe_7_C_3_ case. Note that no changes in the structure of these phases were observed with the increase of the temperature (figure 4). The observation of these phases is surprising and could be attributed to possible changes in the dynamics of CNO nucleation and growth in the presence of the sulfur species (see electronic supplementary material, figures S12 and S13 for EDX analysis in STEM mode). Indeed previous reports have shown that the presence of sulfur may allow the formation of Fe_2_S active areas which would then change the thermodynamics of CNT nucleation. On the basis of the mechanism reported in previous works, we suggest that the sulfur species play a key role also in the nucleation and growth of CNOs by allowing the trapping of these unusual carbide phases within the CNO-core and inhibiting the formation of Fe_3_C [20,21]. This implies also possible intrinsic local changes in the carbon diffusion/extrusion flux dynamics during the CNO nucleation process.

**Figure 5.**
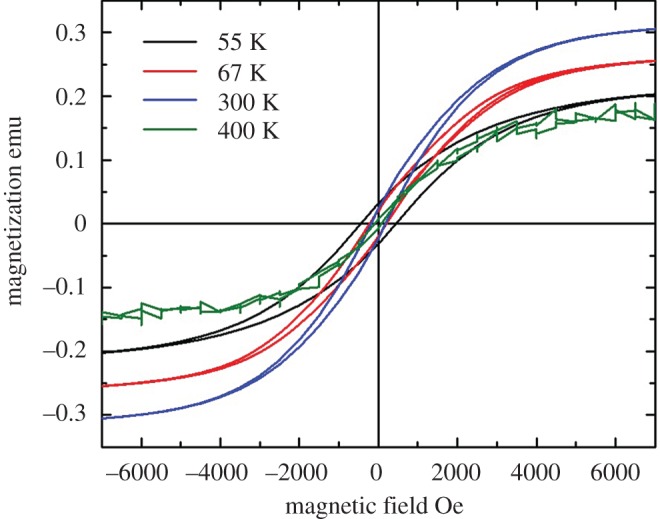
Vibrating sample magnetometry measurement of the as grown CNOs-buckypaper filled with Fe_5_C_2_/Fe_7_C_3_ crystals at different temperatures. The saturation magnetization at 300 K is found to be approximately 47 emu g^−1^.

In the attempt to investigate the magnetic properties of the as grown CNOs, the use of VSM measurements was considered. As shown in figure 5, these measurements confirmed a ferromagnetic-like behaviour of the filled CNOs.

In particular, the saturation magnetization is found to have a peak at around 300 K reaching the value of approximately 47 emu g^−1^. This value of the magnetization is then found to unusually decrease with the temperature, as indicated by the hysteresis loops measured at 67 and 55 K. Such ferromagnetic-like behaviour is then found to collapse at temperatures around 400 K probably due to the proximity of a paramagnetic transition (Curie point).

**Figure 6.**
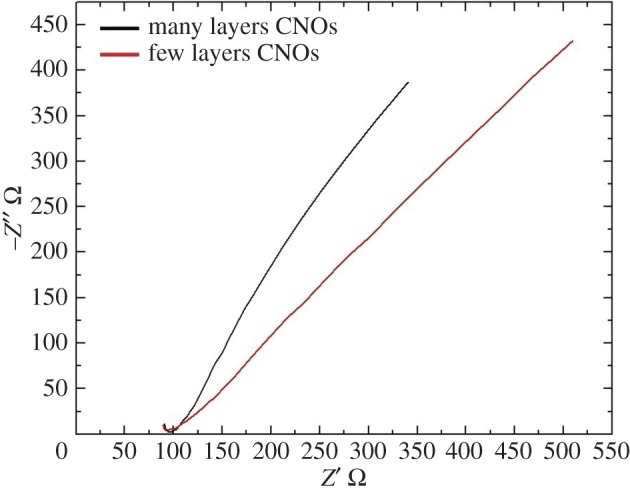
EIS measurement showing in red the property of the as grown few layers CNOs-buckypaper filled with Fe_5_C_2_/Fe_7_C_3_ crystals obtained in the presence of sulfur (red line) and in black the property of the many layers Fe_3_C-filled CNOs grown in presence of only ferrocene.

Additionally, zero field cooling (ZFC) and field cooling (FC) measurements (see electronic supplementary material, figures S3 and S4) clearly show the presence of a not previously reported magnetization peak in the region of 65 K–70 K. The origin of such unusual magnetic behaviour could be associated with possible spin-reorientation dynamics or exchange coupled effects between the Fe_7_C_3_ and Fe_5_C_2_ phases rather than a magnetic phase transition; indeed no magnetic phase transitions are expected for these ferromagnets in such temperature range [17,22]. Note that a previous report on superparamagnetic Fe_7_C_3_ did not show magnetic phase transitions in the temperature range above [17]. Also, a recent report has demonstrated that Fe_5_C_2_ particles with similar dimensions exhibited a ferromagnetic behaviour, therefore, also for this type of phase no magnetic transition is expected in the temperature range above [22]. Note that the (room temperature) magnetic properties reported in this work are also comparable with those of Fe_3_C-filled CNOs where a saturation magnetization of the order of 42 emu g^−1^ was reported [14]. However, Fe_3_C-filled CNOs present a much higher coercivity and saturation magnetization at low temperatures [14] (see also references [23,24] for ZFC and FC studies) due to the absence of exchange coupling phenomena. This latter observation is in agreement with our observations where an unusual decrease in the saturation magnetization is found with the decrease of the temperature. The attention was then focused on the electric properties and performances of the produced CNOs. In particular, in order to have better visualization of these properties, a direct comparison was made between few shell Fe_7_C_3_/Fe_5_C_2_-CNOs and multishell Fe_3_C-CNOs. Figure 6 shows the result of the EIS measurements. Interestingly, the measured curves appear to be characterized by a different slope. Multi-shell Fe_3_C-CNOs appear to exhibit properties compatible with that of pseudo-capacitor-like systems. Instead such behaviour is not observed in few shell Fe_7_C_3_/Fe_5_C_2_-CNOs.

Such a difference in the electrochemical behaviour of these two types of CNOs has to be associated possibly to the much smaller diameter and number of shells within the CNOs produced in this work. Note that a decrease in CNO diameter implies also a possible increase of the curvature and possible re-hybridization effects. Curiously such a dependence on these curvature and diameter parameters was reported also in the case of single-walled CNTs with very small diameters and suggests that with the increase of the curvature and the decrease of the CNO diameter the electric properties of these structures could also be tuned [25] (see also electronic supplementary material, figure S5 for UV-Vis spectroscopy measurement of the as grown CNOs).

## 4. Conclusion

In conclusion, we demonstrated an advanced chemical vapour deposition (CVD) approach in which the use of small quantities of sulfur during the pyrolysis of ferrocene allowed for the control of (i) the diameter of the CNOs, (ii) the number of shells and (iii) the electric properties of these structures. Also, we observed that sulfur induces the formation of unusual carbide phases Fe_5_C_2_/Fe_7_C_3_ within the CNO core which exhibit an unusual spin-reorientation behaviour at around 65–70 K. We demonstrated the morphological, structural, electric and magnetic properties of these new types of CNOs by using SEM, T-XRD, TEM, HRTEM, EIS and T-VSM. Future work will focus on using such sulfur-assisted method for further reduction of the diameter of the CNOs and for possible further manipulation of their electric properties.

## Supplementary Material

Additional data supporting manuscript

## Data accessibility

Details on the sample characterization together with additional figures supporting the findings presented in the manuscript can be found in the electronic supplementary material. Note, this section includes supplementary statistical analyses together with additional structural (XRD), magnetic (ZFC/FC), optical (UV), morphological (TEM) and compositional (STEM and EDX) measurements, respectively. Research materials supporting results in the manuscript can be found also in the online Dryad Digital Repository: http://dx.doi.org/10.5061/dryad.nb8m9 [26].
