# Supplementary material for: Ferromagnetically filled carbon nano-onions: the key role of sulfur in dimensional, structural and electric control
Source: R Soc Open Sci. 2018 Jan 17;5(1):170981. doi: 10.1098/rsos.170981 (PMC5792887; doi:10.1098/rsos.170981)
Supplement: Additional data supporting manuscript [file rsos170981supp1.docx]

Experimental Characterization

SEM investigations were performed with a JSM-7500F at 15 kV. TEM and HRTEM investigations were performed using a 200 kV American FEI Tecnai G^2^F20 fitted with field emission gun. XRD analyses were performed with a Philips Xpert pro MPD (Cu K-α with λ = 0.154 nm) and with a Rigaku Diffractometer in the temperature range of 25-400 degrees ^o^C. In order to identify the phases composing the iron carbide-filled CNOs-buckypaper and extract the unit cell values, the Rietveld refinement method was used. The measurement was fitted with the data cards 1521831 and 1008937 from the Crystallography Open Database. The magnetic measurements were performed by employing a VSM Quantum Design and a VSM Cryogenic Limited London UK.

Electrochemical impedance spectroscopy (EIS) was performed on a CHI660A electrochemical workstation (Shanghai, Chenhua Instrument Co. Ltd., China) in a conventional three-electrode system. A modified electrode, a saturated calomel electrode (SCE) and platinum wire were employed as the working, reference and counter electrode, respectively. EIS measurements were performed in 5 mmol L^-1^ K_3_[Fe(CN)_6_]/K_4_[Fe(CN)_6_] aqueous solution with 0.1 mol L^-1^ KCl as the supporting electrolyte, within the frequency range from 100 kHz to 0.1 Hz, and a bias potential of 0.2 V.

Potassium hexacyanoferrate (II) trihydrate (K_4_[Fe(CN)_6_]·3H_2_O), potassium hexacyanoferrate (III) (K_3_[Fe(CN)_6_]) and potassium chloride (KCl) were provided by Sinopharm Chemical Reagent Co. Ltd. (Shanghai, China). The chemicals were used without further purification.

UV-Vis absorption spectra was recorded using a Hanon i5 UV-Vis Spectrophotometer (Jinan Hanon Instruments Co., Ltd., China)


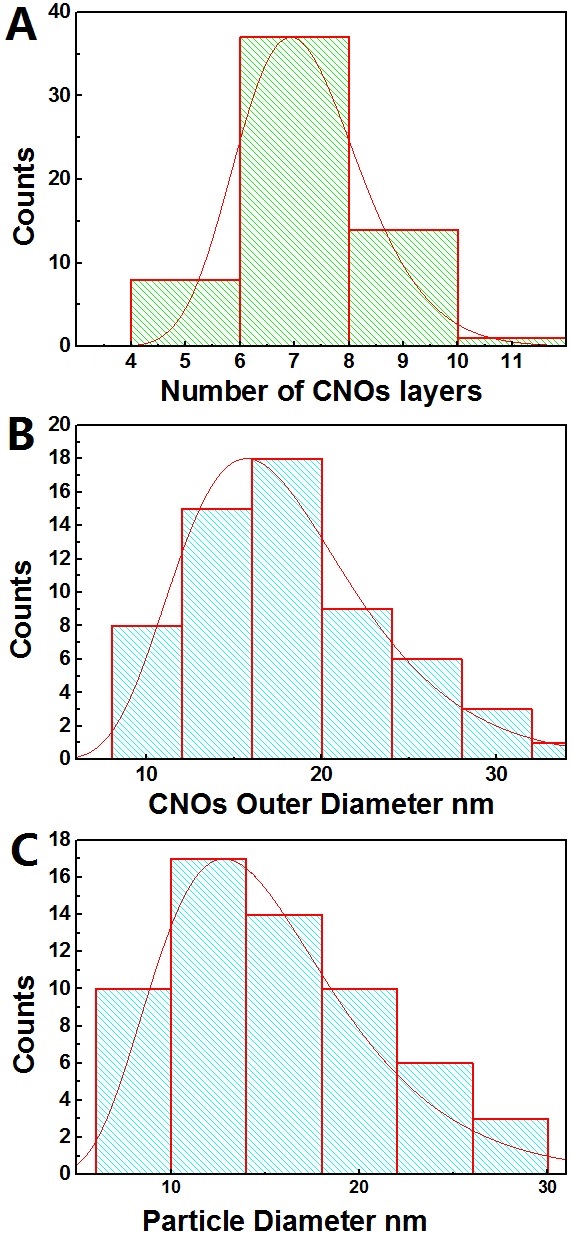


Fig. 1 Supp. Info.: Statistical analyses of the as grown CNOs filled with Fe_7_C_3_/Fe_5_C_2_ crystals by pyrolysis of 400 mg of ferrocene and 2.5 mg of sulfur


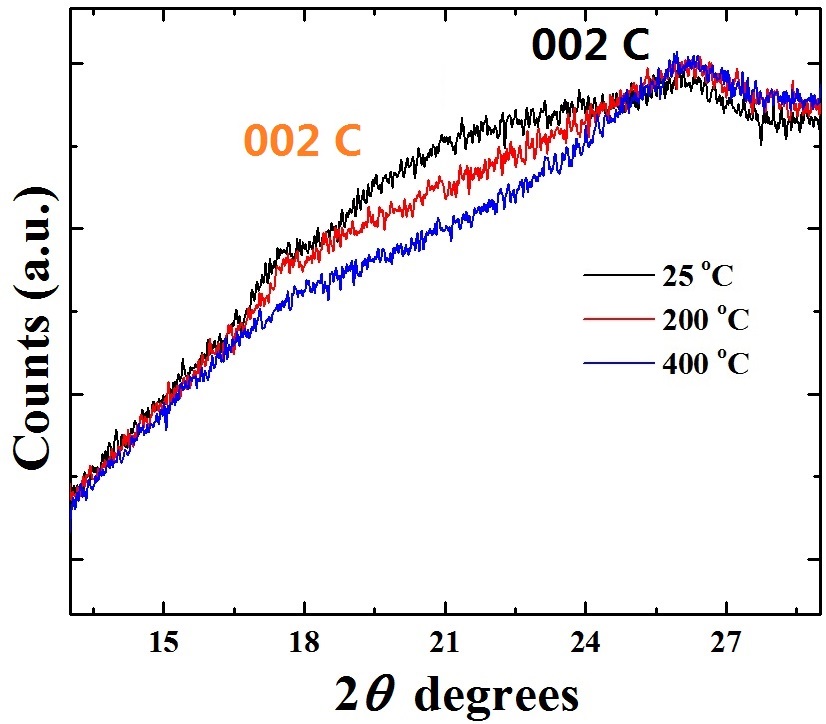


Fig. 2 Supp. Info.: XRD diffractogram of the as grown CNOs filled with Fe_7_C_3_/Fe_5_C_2_ crystals by pyrolysis of 400 mg of ferrocene and 2.5 mg of sulfur showing the graphitic arrangement of the CNOs. The measurements were performed at the temperature of 25 ^o^C, 200 ^o^C and 400 ^o^C.

.:
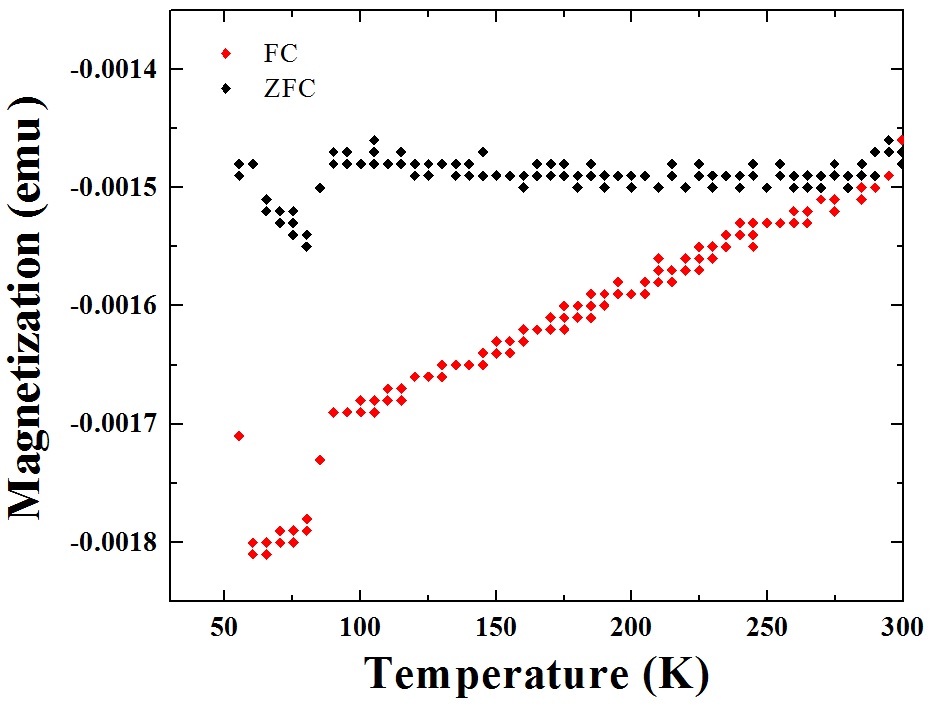


Fig. 3 Supp. Info.: ZFC and FC (at the field of 300 Oe) measurements of the as grown CNOs filled with Fe_7_C_3_/Fe_5_C_2_ crystals by pyrolysis of 400 mg of ferrocene and 2.5 mg of sulfur. Note that these measurements were performed with a small quantity of CNOs, 0.9 mg. A clear transition appears to be present in the region of 60-75 K. Note that the negative value of magnetization can be associated to the low weight of the measured sample.


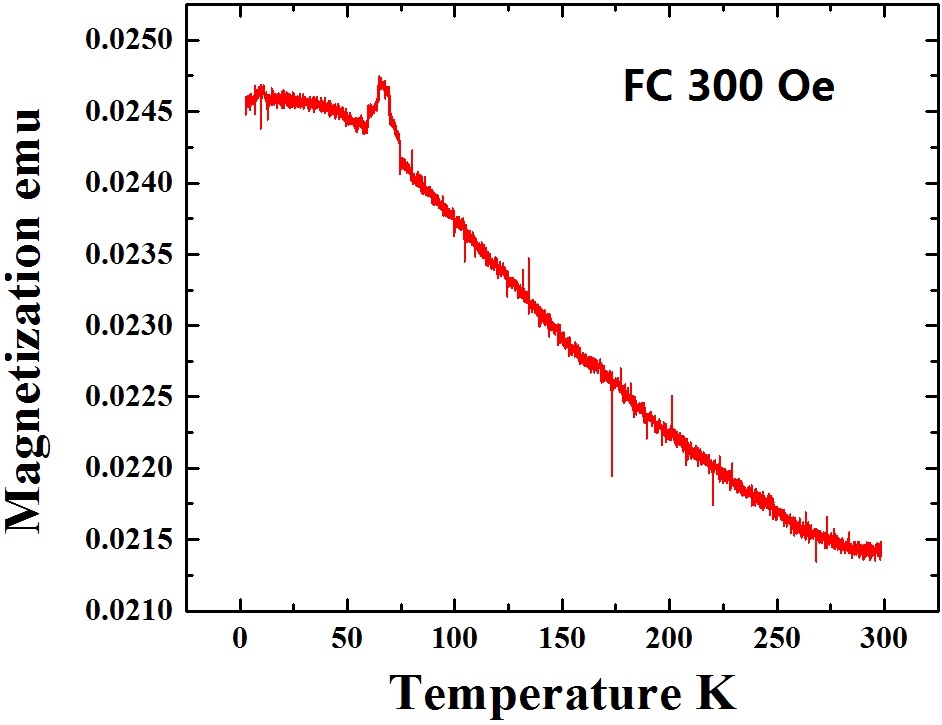


Fig. 4 Supp. Info.:FC measurements of the as grown CNOs filled with Fe_7_C_3_/Fe_5_C_2_ crystals by pyrolysis of 400 mg of ferrocene and 2.5 mg of sulfur. Note that these measurements were performed with larger quantity of CNOs, approximately 6 mg. A clear transition appears to be present in the region of 60-75 K.


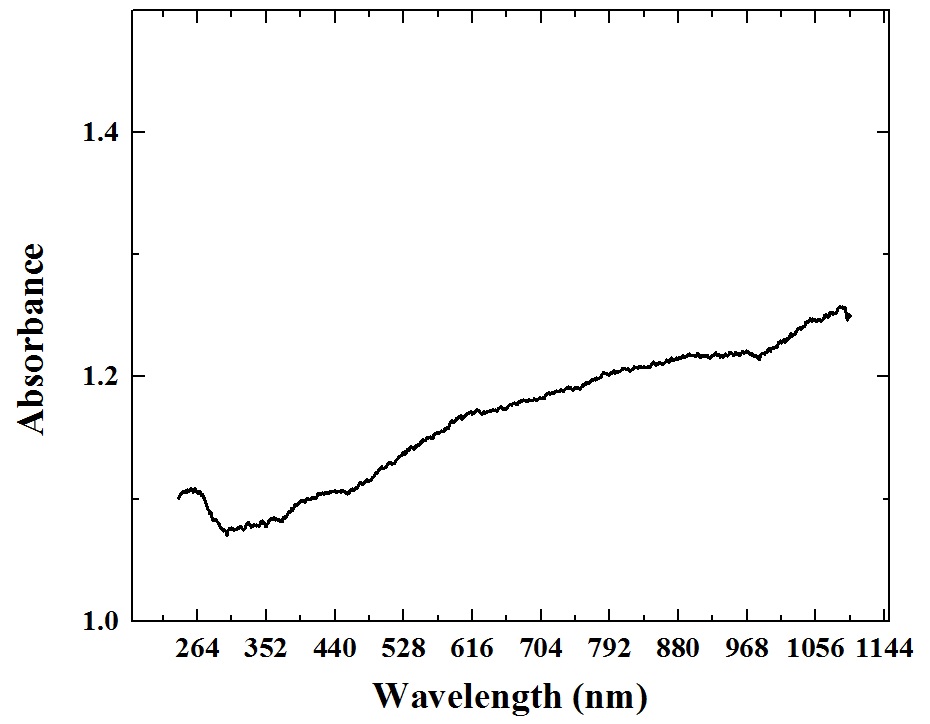


Fig. 5 Supp. Info.: UV Spectroscopy measurement of the as grown CNOs filled with Fe_7_C_3_/Fe_5_C_2_ crystals by pyrolysis of 400 mg of ferrocene and 2.5 mg of sulfur. No peak is found, suggesting that no band gap is present in the electronic structure of the CNOs.


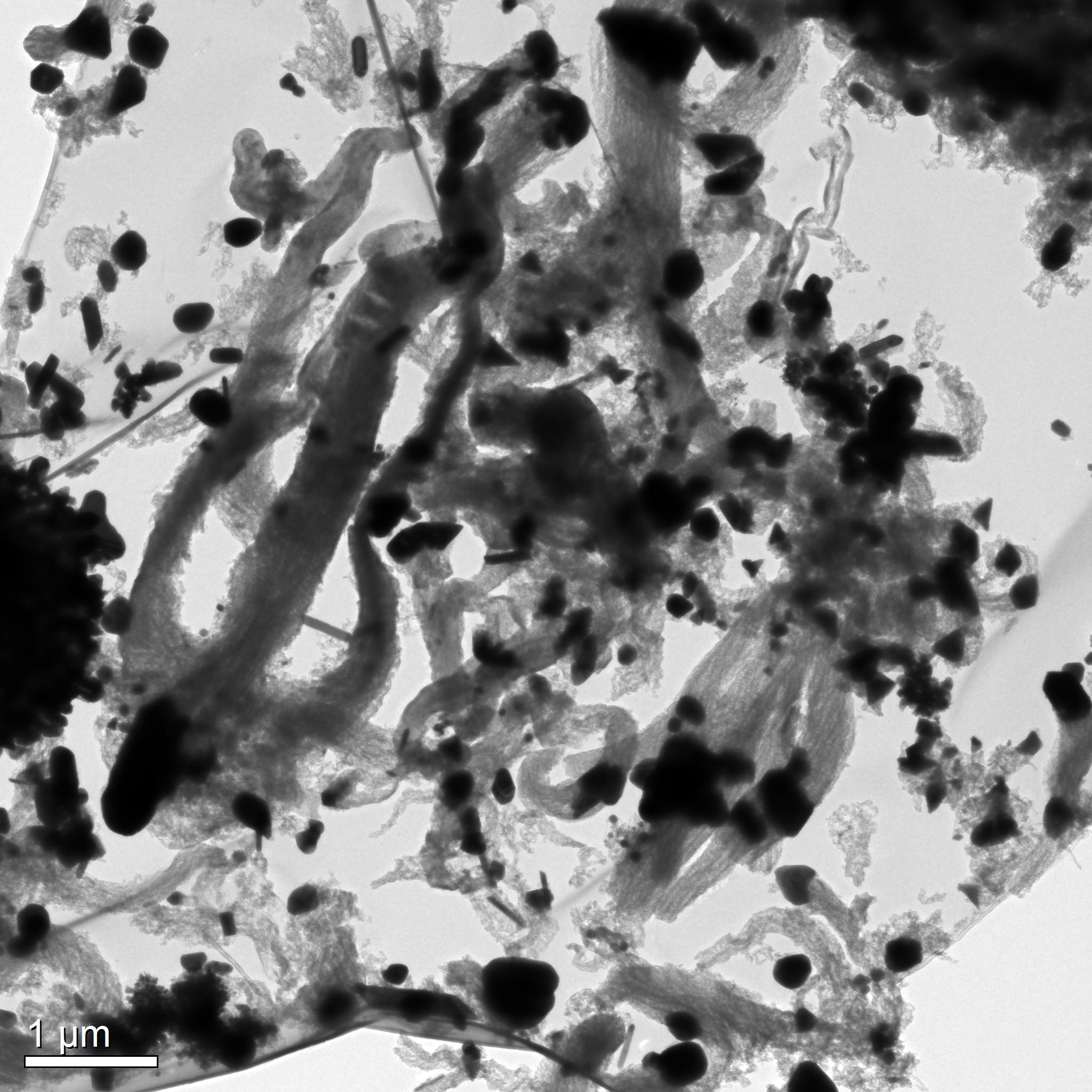


Fig. 6 Supp. Info.: TEM micrograph showing the CNOs-like structures obtained by sublimation and pyrolysis of 600 mg of Ferrocene and 2.5mg of Sulfur (see experimental info for synthesis condition details). The obtained structures appear to be arranged in unusual shapes with an octopus-like morphology.


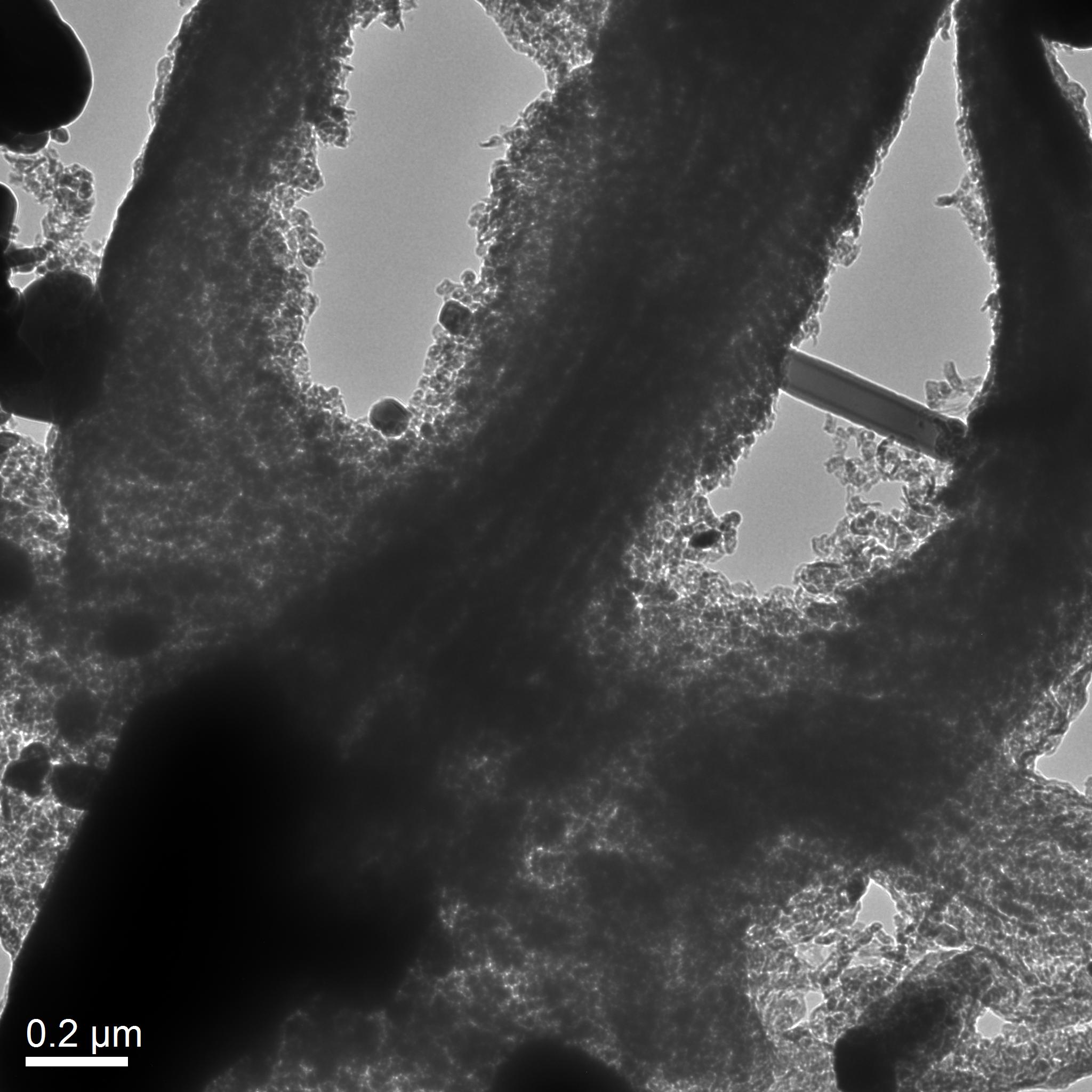


Fig. 7 Supp. Info.: TEM micrograph showing the CNOs-like structures obtained by sublimation and pyrolysis of 600 mg of Ferrocene and 2.5mg of Sulfur (see experimental info for synthesis condition details). Detail of the octopus-like structures, showing turbostratic carbon composition.


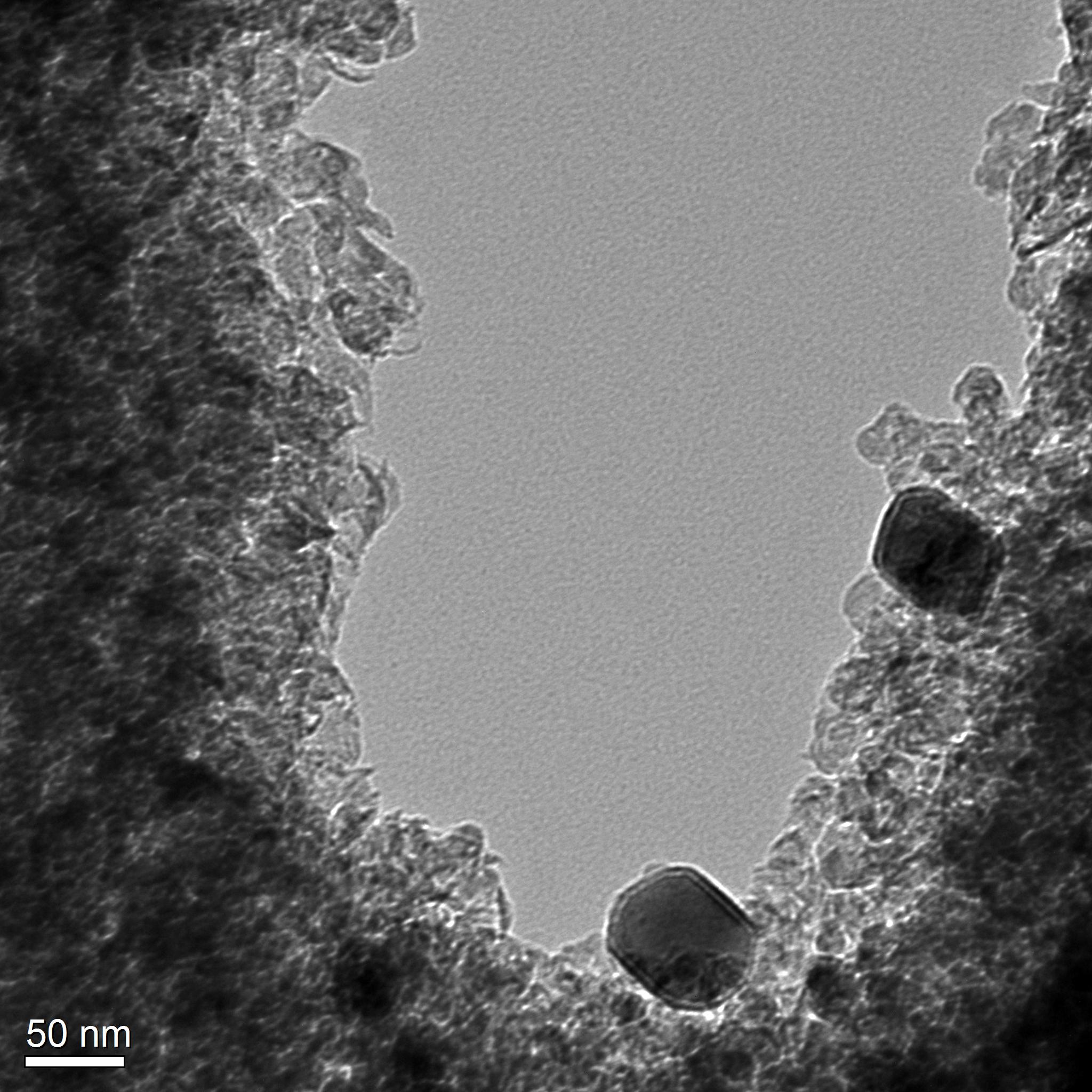


Fig. 8 Supp. Info.: TEM micrograph showing the CNOs-like structures obtained by sublimation and pyrolysis of 600 mg of Ferrocene and 2.5mg of Sulfur (see experimental info for synthesis condition details). Detail showing a mixture of carbon flake-like structures and filled CNOs.


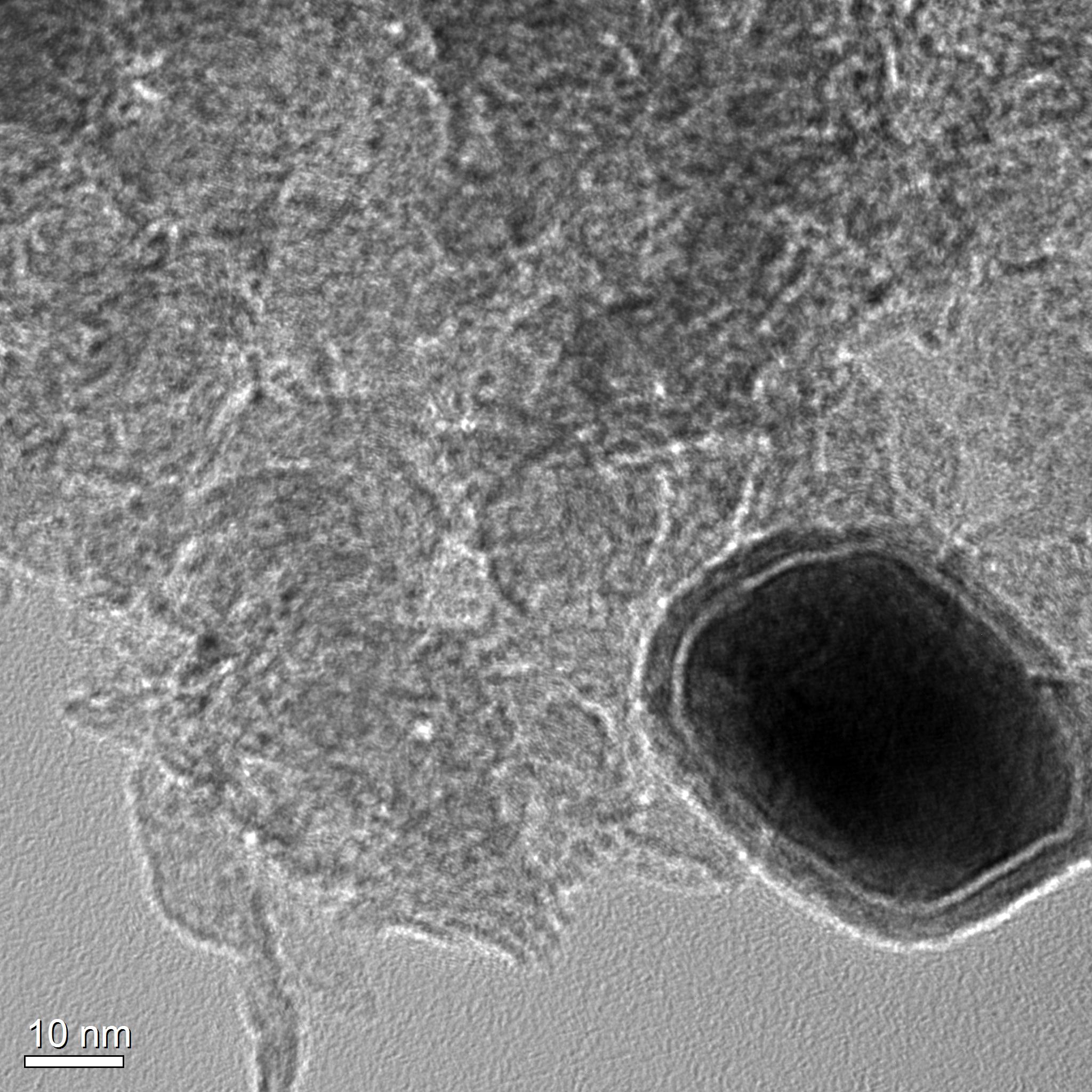


Fig. 9 Supp. Info.: TEM micrograph showing with higher detail the CNOs-like and carbon flake-like structures obtained by sublimation and pyrolysis of 600 mg of Ferrocene and 2.5 mg of Sulfur (see experimental info for synthesis condition details).


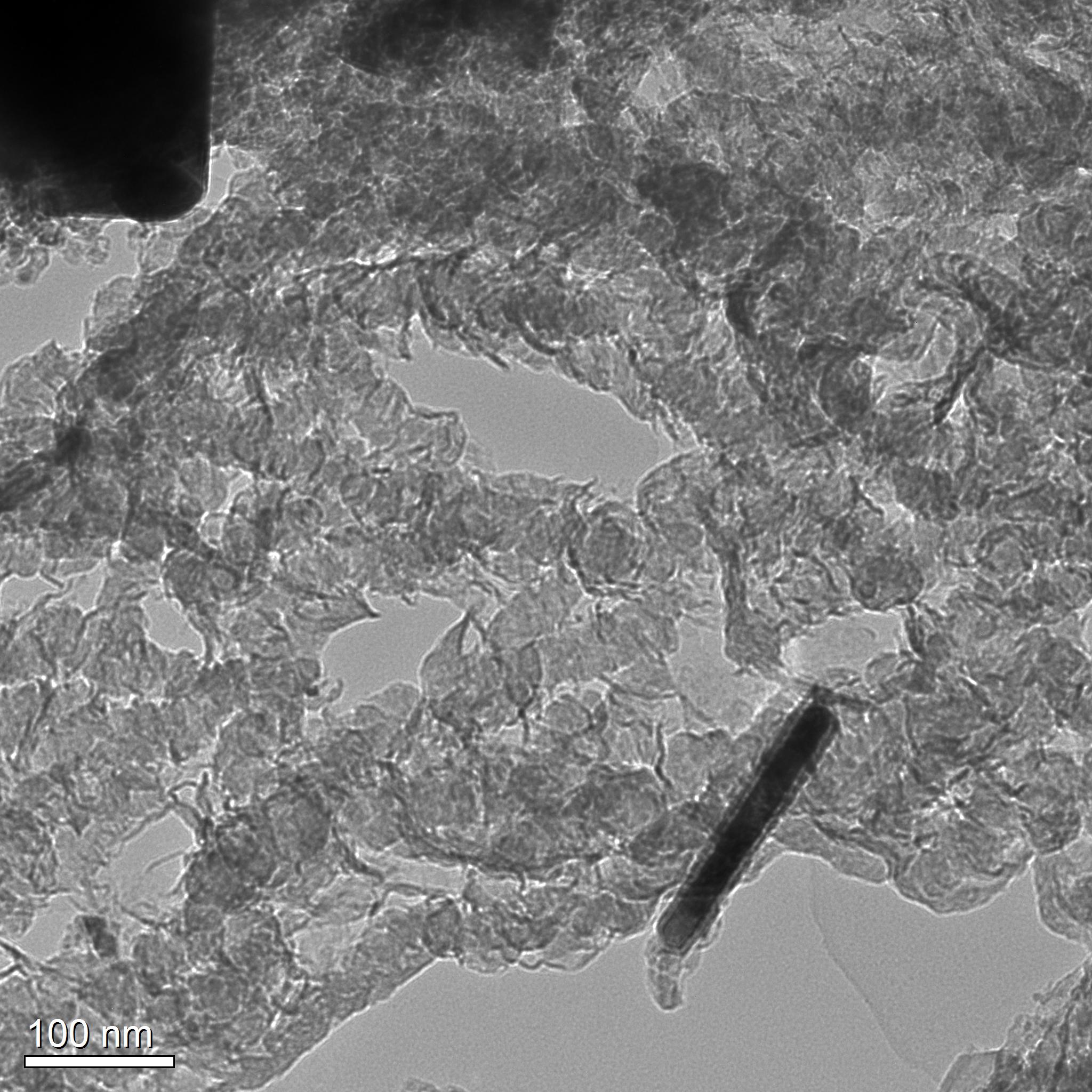


Fig. 10 Supp. Info.: TEM micrograph showing more examples of elongated CNOs-like and carbon flake-like structures obtained by sublimation and pyrolysis of 600 mg of Ferrocene and 2.5mg of Sulfur (see experimental info for synthesis condition details).


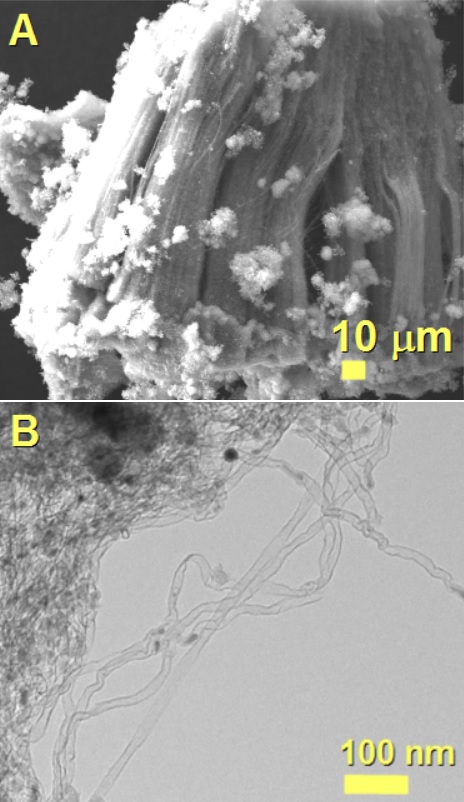


Fig. 11 Supp. Info.: SEM (A) and TEM (B) micrographs showing the morphology of a flake of the as grown single wall CNTs obtained in the Si/SiO_2_ substrate by the pyrolysis of 400 mg of ferrocene and 2.5 mg of sulfur.


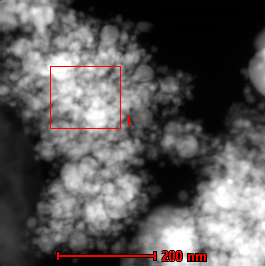


Fig. 12 Supp. Info.: STEM micrograph of the as grown CNOs filled with Fe7C3/Fe5C2 crystals by pyrolysis of 400 mg of ferrocene and 2.5 mg of sulfur. Note that STEM provides details related to the atomic contrast in the sample, therefore the heavier elements will appear brighter in the image (Fe in this case).


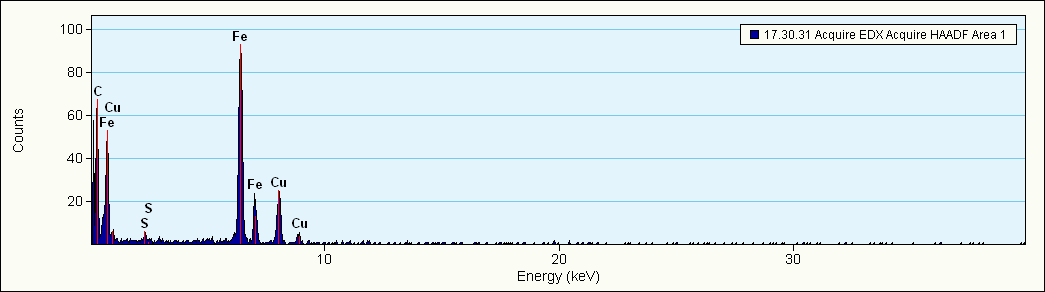


Fig. 13 Supp. Info.: EDX analyses of the area indicate by the red square in Fig.12 Supp.Info. The following elemental atomic % were measured: 16.95% of Fe, 0.32 % of S, 82.71% of C. The following weight % were measured: 48.53% of Fe, 0.53% of S, 50.92% of C.
